# Astrocytes—The Ultimate Effectors of Long-Range Neuromodulatory Networks?

**DOI:** 10.3389/fncel.2020.581075

**Published:** 2020-09-29

**Authors:** Anthony G. Pacholko, Caitlin A. Wotton, Lane K. Bekar

**Affiliations:** Department of Anatomy, Physiology, and Pharmacology, University of Saskatchewan, Saskatoon, SK, Canada

**Keywords:** neuromodulator, gliotransmitter, brain-state, Na^+^/K^+^-ATPase, potassium homeostasis in brain, inward rectifier (channel), cortical oscillations and functional connectivity

## Abstract

It was long thought that astrocytes, given their lack of electrical signaling, were not involved in communication with neurons. However, we now know that one astrocyte on average maintains and regulates the extracellular neurotransmitter and potassium levels of more than 140,000 synapses, both excitatory and inhibitory, within their individual domains, and form a syncytium that can propagate calcium waves to affect distant cells *via* release of “gliotransmitters” such as glutamate, ATP, or adenosine. Neuromodulators can affect signal-to-noise and frequency transmission within cortical circuits by effects on inhibition, allowing for the filtering of relevant vs. irrelevant stimuli. Moreover, synchronized “resting” and desynchronized “activated” brain states are gated by short bursts of high-frequency neuromodulatory activity, highlighting the need for neuromodulation that is robust, rapid, and far-reaching. As many neuromodulators are released in a volume manner where degradation/uptake and the confines of the complex CNS limit diffusion distance, we ask the question—are astrocytes responsible for rapidly extending neuromodulator actions to every synapse? Neuromodulators are known to influence transitions between brain states, leading to control over plasticity, responses to salient stimuli, wakefulness, and sleep. These rapid and wide-spread state transitions demand that neuromodulators can simultaneously influence large and diverse regions in a manner that should be impossible given the limitations of simple diffusion. Intriguingly, astrocytes are ideally situated to amplify/extend neuromodulator effects over large populations of synapses given that each astrocyte can: (1) ensheath a large number of synapses; (2) release gliotransmitters (glutamate/ATP/adenosine) known to affect inhibition; (3) regulate extracellular potassium that can affect excitability and excitation/inhibition balance; and (4) express receptors for all neuromodulators. In this review article, we explore the **hypothesis** that astrocytes extend and amplify neuromodulatory influences on neuronal networks *via* alterations in calcium dynamics, the release of gliotransmitters, and potassium homeostasis. Given that neuromodulatory networks are at the core of our sleep-wake cycle and behavioral states, and determine how we interact with our environment, this review article highlights the importance of basic astrocyte function in homeostasis, general cognition, and psychiatric disorders.

## Introduction

Acetylcholine (ACh) from the basal forebrain, dopamine (DA) from the ventral tegmental area/substantia nigra, histamine (HA) from the tuberomammillary nucleus, norepinephrine (NE) from the locus coeruleus, and serotonin (5HT) from the raphe nuclei can all be released in a volume transmission fashion to exert their neuromodulatory influence over large areas of the brain simultaneously (Zoli and Agnati, 1996; Zoli et al., 1998; Fuxe et al., 2010). It is estimated that less than 20% of these neuromodulatory system varicosities form conventional synapses (Séguéla et al., 1990; Cohen et al., 1997; Descarries and Mechawar, 2000; Mechawar et al., 2000; Descarries et al., 2004), with the remainder releasing neuromodulators in a volume fashion to exert effects on axons, dendrites, astrocytes, microglia, and blood vessels. It is even postulated that astrocytes and astrocyte function are a major target of this volume transmission (Hirase et al., 2014; Fuxe et al., 2015). Given the known ability for neuromodulators to rapidly regulate transitions in brain state/behavior (Lee and Dan, 2012) and that diffusion of neuromodulators is limited by inefficiencies imposed by the confines of the extracellular space, reuptake transporters, enzymatic breakdown, and astrocyte processes (Syková and Nicholson, 2008; Syková and Vargová, 2008), a simple question remains: How do neuromodulators affect such rapid, wide-spread changes in brain-state and behavior? Given the discovery of the increasingly complex role astrocytes play in synaptic function, this review article explores the potential that astrocytes serve to extend and possibly amplify neuromodulatory actions.

## Astrocytes Are Ideally Suited to Modulate Every Synapse and Synchronize Network Activity

**Figure 1:**
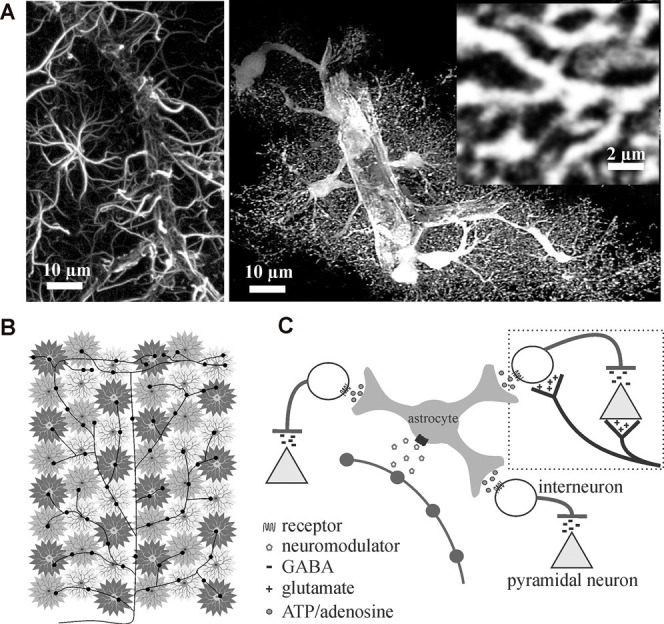
Astrocytes are ideally situated to extend neuromodulators’ influence over cortical inhibition to multiple synapses. **(A)** Traditional glial fibrillary acidic protein (GFAP) immunolabeling in the rat cortex (left image) only outlines major processes giving them their star-like shape. In contrast, cytosolic enhanced green fluorescence protein expression in astrocytes reveals their highly complex and delicate morphology *in situ* (right image). Astrocytes consist of numerous fine processes that form a matrix-like substrate (inset) that is in close apposition to axons, dendrites, synapses, and cell bodies. Images adapted from Simard and Nedergaard (2004) with permission. **(B,C)** Conceptual representation of a single locus coeruleus norepinephrine neuron, projecting from the corpus callosum through to the surface of the cortex, illustrating how volume release can affect individual astrocyte regulated synaptic islands for rapid and wide-spread effect.

Astrocytes are believed to outnumber neurons in the mammalian cortex (Khakh and Sofroniew, 2015) and, under normal conditions, are spaced such that processes do not overlap (Bushong et al., 2002, 2004; Oberheim et al., 2006; Nimmerjahn and Bergles, 2015). The larger glial fibrillary acidic protein (GFAP)-positive processes easily observed by immunohistochemical methods only label 10–20% of the astrocyte volume. Eighty to ninety percent of astrocyte membrane volume is made up of ultrathin processes and protrusions (Bushong et al., 2002). Thus, astrocytes are less of a “star” shape, as their name suggests based on original GFAP immunochemistry, and more like a sponge forming part of the matrix within which blood vessels, neurons, axons, and synapses are embedded (Figure 1A). Interestingly, in addition to their well-established homeostatic roles, the ultrathin perisynaptic astrocytic processes physically interact with synapses in a dynamic fashion (Reichenbach et al., 2010; Ghézali et al., 2016) that is dependent on activity-induced plasticity signals; supporting a role for astrocytes in physical synapse stabilization (Bernardinelli et al., 2014; Ghézali et al., 2016). It has been proposed that all synapses within an individual astrocytes’ domain (>140,000) are controlled by the homeostatic features and gliotransmitter milieu of that single astrocyte, forming units that a single astrocyte can modulate and synchronize termed a “Synaptic Island” (Halassa et al., 2007). Thus, the different brain regions can be divided into these equally spaced synaptic islands that are shaped by individual astrocyte morphologies.

In addition to physically parsing the brain into functional synaptic islands, astrocytes are characterized by their highly negative resting membrane potentials (near the K^+^ equilibrium potential), high K^+^ permeability, and extensive gap junctional coupling. The high baseline K^+^ conductance and extensive gap junctional coupling minimize activity-induced astrocyte membrane potential fluctuations, giving the connected astrocyte syncytium “isopotentiality” (Ma et al., 2016; Kiyoshi et al., 2018). Coupling of any given astrocyte to between seven and nine nearest neighbors with coupling resistance lower than membrane input resistance, allows rapid short-circuiting of any activity-induced changes in astrocyte membrane potential (Ma et al., 2016; Kiyoshi et al., 2018). This gives the astrocyte syncytium the ideal characteristics for uptake and redistribution of extracellular K^+^ and—when these characteristics are modulated—ultimate control of neural excitability (discussed in detail below). Additionally, in contrast to individual astrocyte modulation and potential synchronization of their individual synaptic islands through local gliotransmission, the astrocyte syncytium controls extracellular K^+^ on the much larger multi-“island” scale for potential synchronization across whole networks and entire brain regions (Kiyoshi et al., 2018); ideally suited for modulating brain state.

Accumulating evidence suggests astrocytes are intimately involved in brain state transitions. It is believed that astrocytes do this *via* the release of gliotransmitters (Poskanzer and Yuste, 2011, 2016; Deemyad et al., 2018) and/or regulation of extracellular potassium (Wang et al., 2012a,b; Ding et al., 2016; Rasmussen et al., 2019). Many types of neurons including cortical pyramidal neurons are known to possess intrinsic ion channel properties that enable neuron cycling between two major states termed “UP”-state (or activated state) and “DOWN”-state (or resting state) that are associated with membrane depolarization and hyperpolarization, respectively. Influencing which membrane state the neurons are in governs which neurons are actively engaged within vast neural networks that can span the whole brain. Spontaneous or stimulated astrocyte calcium events in acutely isolated brain slices increase synchronized neuron UP states (Poskanzer and Yuste, 2011; Pirttimaki et al., 2017) that may represent astrocyte-induced neocortical slow oscillations seen under similar conditions *in vivo* (Poskanzer and Yuste, 2016). The shift in the neuronal state was dependent on extracellular glutamate accumulation/release and involved a shift in ATP/adenosine signal balance. Although glutamatergic and purinergic signaling was involved, the effects of changes in extracellular potassium were not experimentally ruled out (Poskanzer and Yuste, 2011, 2016). Studies in cerebellar Purkinje neurons, also known to cycle between UP and DOWN states (bistability), demonstrate that astrocyte calcium responses mediate uptake of extracellular K^+^ to affect bistability, resulting in an enhanced output of Purkinje neurons in the UP state (Wang et al., 2012a). Such a mechanism would also fit experimental findings in the cortex ascribed to glutamate and purines (Poskanzer and Yuste, 2011, 2016) and, although a recent study showed that changes in extracellular K^+^ parallel brain state transitions (Rasmussen et al., 2019), indirectly implicating astrocytes, this still needs to be addressed directly.

Given that each astrocyte ensheathes and maintains over 140,000 synapses within its individual domain (Bushong et al., 2002) and can sense single synaptic events (Di Castro et al., 2011; Panatier et al., 2011), astrocytes are perfectly positioned to communicate with many excitatory and inhibitory synapses rapidly. Add to this the fact that astrocytes are known to possess receptors for the different neuromodulators (Porter and McCarthy, 1997) that are involved in regulating brain state (Lee and Dan, 2012), it can be reasoned that astrocytes are in the perfect position to rapidly affect excitability and extend neuromodulator effects across large networks. A single locus coeruleus norepinephrine axon extending from corpus callosum through to the cortical surface with multiple varicosity release sites can theoretically recruit many astrocyte-controlled synaptic islands to affect millions of synapses and excitation/inhibition balance (Figures 1B,C).

## Extracellular K^+^ Can Dynamically Impact Network Activity

Astrocytes are well-known for maintaining the concentration of extracellular potassium ([K^+^]_e_) *via* both passive and active uptake and redistribution. This process primarily involves inward rectifying K^+^ channels (Kir4.1 specifically; Chever et al., 2010; Sibille et al., 2015) and the Na^+^/K^+^ ATPase (Larsen et al., 2014, 2016a; Stoica et al., 2017). Although redistribution to regions of lower [K^+^]_e_ may not necessitate coupling within the astrocyte syncytium, the extensive coupling in conjunction with high K^+^ conductance enables astrocytes to maintain a highly negative resting membrane potential and minimize potential changes in the face of local changes in [K^+^]_e_. Thus, as local activity-induced [K^+^]_e_ increases—shifting the K^+^ equilibrium potential (E_K_) more positive than the astrocyte syncytium isopotential (E_m_)—K^+^ flows into astrocytes locally through the Kir4.1 channel (E_K_ > E_M_). At more distant sites, the mild depolarization of the astrocyte syncytium membrane isopotential without any change in E_K_ results in K^+^ efflux (E_K_ < E_M_), completing the activity-induced redistribution of K^+^ from areas of high to areas of low [K^+^]_e_. In addition to this passive movement of K^+^, active uptake *via* the Na^+^/K^+^ ATPase can occur under conditions of high-frequency synaptic activity (reviewed in Larsen et al., 2016a) and/or evoked astrocyte calcium responses that drive Na^+^/K^+^ ATPase activity *via* increased intracellular Na^+^ by way of the Na^+^/Ca^2+^ exchanger (Wang et al., 2012b). As both Kir4.1 and Na^+^/K^+^ ATPase are known to be regulated by various intracellular messengers, any neuromodulator that regulates Kir4.1 or Na^+^/K^+^ ATPase activity may result in altered [K^+^]_e_ with direct influence on network behavior.

Neuronal excitability is under the direct influence of [K^+^]_e_-mediated effects on resting membrane potential that influence excitation/inhibition balance and gain modulation. Small decreases in extracellular potassium (<1 mM; increased driving force for K^+^ to leave the cell) leads to mild hyperpolarization of neurons with a resulting suppression of miniature and spontaneous excitatory postsynaptic potentials (mEPSPs/sEPSPs) with no effect on evoked EPSPs (eEPSPs) or miniature inhibitory postsynaptic potentials (mIPSPs; Wang et al., 2012b). The hyperpolarization is thought to decrease the probability of glutamate release and the negative shift in E_K_ reduces the amplitude/duration of AMPA receptor (permeable to Na^+^ and K^+^)-mediated depolarizations. The result is an associated increase in signal-to-noise (no change in eEPSPs, a decrease in sEPSPs, and no effect on mIPSPs; Wang et al., 2012b). Interestingly, a similar shift in excitation/inhibition balance (also favoring inhibition) is observed when extracellular K^+^ is mildly increased (1–2 mM from ~3.5 to 5.5 mM; well below typical ceiling level of 10–12 mM in the cortex). The K^+^-mediated depolarization increases the frequency of spontaneous excitatory activity, but reduces action potential amplitude, *via* slowing of sodium channel recovery from inactivation (Meeks and Mennerick, 2004), impacting both spontaneous and evoked excitatory amplitudes. Increased [K^+^]_e_ was also found to depolarize interneurons increasing their spontaneous activity (Shin et al., 2010). While increased [K^+^]_e_ affects both excitatory and inhibitory neuronal activity, the faster firing frequency of interneurons, due to the expression of Kv3 channels (Boddum et al., 2017; Ferguson and Gao, 2018), shifts the balance toward inhibition as [K^+^]_e_ rises. Thus, as [K^+^]_e_ decreases, some aspects of excitation decrease while inhibition remains unchanged resulting in an increase in signal-to-noise whereas, as [K^+^]_e_ increases, amplitudes of EPSPs decrease and frequency of inhibitory activity increase for a general suppression of activity. Interestingly, elevated [K^+^]_e_ and resulting cortical depolarization has recently been shown *in vivo* to be associated with the onset of locomotion (change in brain state) with an accompanying increase in visual gain modulation (Rasmussen et al., 2019). Thus, both excitation-inhibition balance and gain of network activity appears to be under tight influence of [K^+^]_e_.

**Figure 2:**
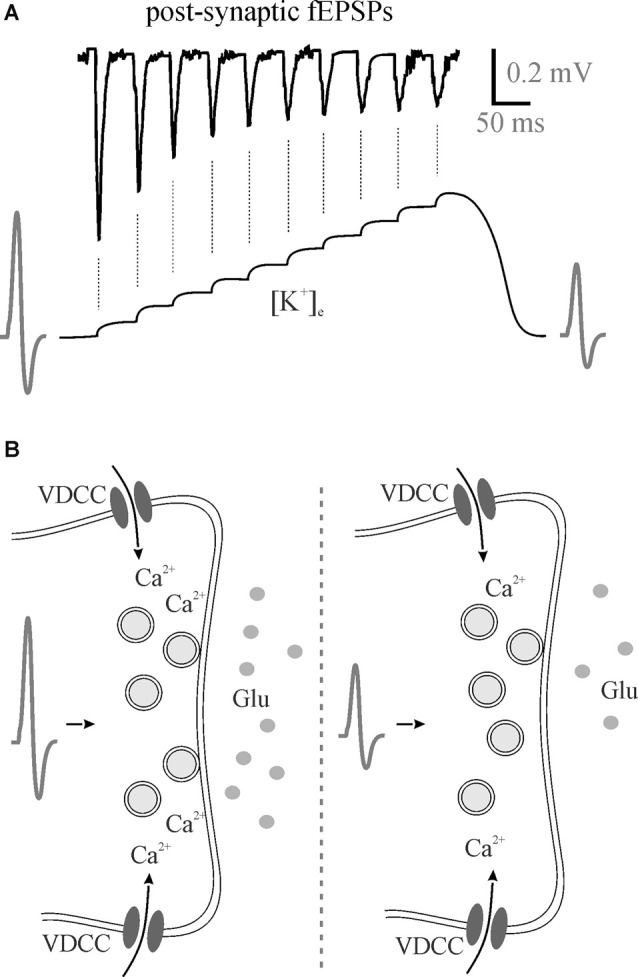
Perisynaptic K^+^ buildup can decrease neurotransmitter release. **(A)** 50 Hz stimulation trains lead to the accumulation of extracellular K^+^ that theoretically depolarizes membranes reducing presynaptic action potentials with a subsequent reduction in neurotransmitter release as evident by post-synaptic fEPSPs. **(B)** A hypothesized reduction in action potential amplitude means less Ca^2+^ entry through voltage-gated calcium channels for less vesicular neurotransmitter release. fEPSPs, field excitatory postsynaptic potentials; Glu, glutamate; VDCC, voltage-gated calcium channel.

In addition to effects on excitation-inhibition balance and gain modulation, [K^+^]_e_ affects frequency transmission and brain state. In our most recent study, we found that decreasing perfusate [K^+^] reduced somatosensory adaptation similar to 5HT and NE (Wotton et al., 2020). During the repetitive firing of action potentials, [K^+^]_e_ increases at the synapse with each subsequent stimulation (Figure 2A). The most likely scenario to account for the reduction in EPSP amplitude would be that the depolarization of the pre-synaptic terminal, as the [K^+^]_e_ rises, and slowing of sodium channel recovery from inactivation (Meeks and Mennerick, 2004) leads to a reduction in action potential amplitude, decrease in voltage-gated calcium channel opening and reduced neurotransmitter release (Figure 2B). Accordingly, in the low [K^+^]_e_ perfusate, K^+^ does not accumulate to the same extent and less frequency adaptation is evident mimicking 5HT- and NE-mediated effects on K^+^ clearance (Wotton et al., 2020). In light of this, the regulation of activity-dependent [K^+^]_e_ increases *via* both passive and active astrocyte uptake mechanisms enables rapid and precise control over somatosensory frequency transmission. Potassium accumulation can also have a broader impact than frequency transmission within a single synapse. A recent study using the pharmacological disruption of astrocyte inward rectifiers or gap junction coupling demonstrated an important role for astrocyte K^+^ regulation in cortical oscillations (Bellot-Saez et al., 2018). By disrupting K^+^ uptake or astrocyte coupling they showed an increase in [K^+^]_e_ that was associated with an increase in power of multiple high frequency oscillations. This is consistent with another recent study showing that elevation in [K^+^]_e_ in the cortex *in vivo* increases the power of high frequency oscillations as well as increases gain in the visual cortex (Rasmussen et al., 2019).

## Gliotransmitters Affect Both Excitation and Inhibition

Similar to how controlling [K^+^]_e_ can influence synaptic activity, astrocytes can also release the gliotransmitters glutamate and D-serine to affect synaptic function. Computational modeling suggests astrocytic glutamate release disrupts the occurrence of high-frequency post-synaptic firing (Flanagan et al., 2018) and both short- and long-term plasticity (De Pittà and Brunel, 2016). Extra synaptic activation of glutamate receptors in the pre-synapse by astrocyte-originating glutamate can either increase or decrease the probability of release for neurotransmitters (Santello and Volterra, 2009; De Pittà and Brunel, 2016). This glutamate-dependent effect influences synaptic activity (Fiacco and McCarthy, 2004; Jourdain et al., 2007) at both excitatory and inhibitory synapses (Liu et al., 2004; Jourdain et al., 2007; Perea and Araque, 2007; Benedetti et al., 2011). Alternatively, D-serine released in a Ca^2+^-dependent manner from astrocytes is also associated with synaptic function; namely plasticity (Henneberger et al., 2010). It has also been associated with inhibition of GABAergic excitability (Wu et al., 2017). Although D-serine-mediated gliotransmission was called into question by data that suggested neurons were the primary source of D—serine production and release (Wolosker et al., 2016), this was subsequently clarified (Papouin et al., 2017). Glutamate and D-serine have the potential to be participants in the astrocyte-mediated extension of neuromodulatory effects.

In addition to glutamate and D-serine, the purines ATP and adenosine are gliotransmitters that can also extend neuromodulator effects. Both ATP and adenosine can modulate excitation through their respective receptors. P2Y receptors, for ATP, are more physiological than their P2X counterparts (Khakh, 2001) and are predominantly associated with a reduction of excitatory activity and are involved in heterosynaptic depression (Koizumi et al., 2003; Zhang et al., 2003; Pascual et al., 2005; Hussl and Boehm, 2006). This occurs through the inhibition of calcium channels or increased activation of outward K^+^ currents (Hussl and Boehm, 2006). Generally, adenosine decreases or increases excitatory activity through A1 and A2A receptors, respectively (Newman, 2003; Fontanez and Porter, 2006; Fredholm et al., 2007; Rebola et al., 2008; Panatier et al., 2011). Both ATP and adenosine are also intricately associated with effects on inhibition. The P2Y1 receptor is demonstrated to be particularly important in increasing interneuron activity. For example, it was shown that astrocytic ATP activation of P2Y1 receptors on hippocampal interneurons increased cation currents and simultaneously decreased K^+^ currents; increasing the activity of interneurons and release of GABA which inhibited the downstream neurons (Bowser and Khakh, 2004). Additionally, P2Y1 activation on interneurons led to both short- and long-term increases in spontaneous and evoked inhibitory GABA_A_ currents in the cerebellum (Saitow et al., 2005). More specifically, sIPSC amplitude and frequency were both increased rapidly, while a stimulus-evoked IPSC increase was observed ~20 min following the P2Y1 activation. Adenosine, too, is implicated in modulating inhibitory synapses. Similar to A1 effects on excitatory neurons, there is evidence that A1 receptors decrease the activity of the inhibitory synapses in the thalamocortical pathway (Fontanez and Porter, 2006). A2A receptor activation, however, in the hippocampus is associated with increased activity of select GABAergic interneurons (Rombo et al., 2015), and in the tuberomammillary nucleus, A2A activation was associated with GABA release and induction of sleep (Hong et al., 2005). In brief, purinergic gliotransmitters are important modulators of synaptic excitation and inhibition; much like the neuromodulators we hypothesize act through astrocytes.

## Neuromodulators Gate Shifts in Brain State for Optimal Performance

**Figure 3:**
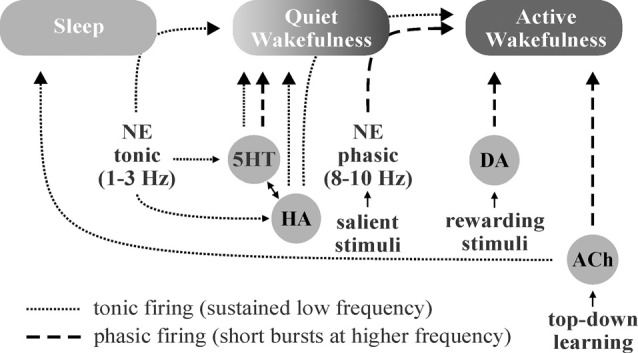
Schematic illustration of putative neuromodulator roles in the regulation of sleep-wake states. Norepinephrine (NE)-, histamine (HA)-, and serotonin (5HT)-associated neurons are quiet-to-quiescent during sleep and tonically active during wakefulness. NE appears to be chiefly involved in rousing from sleep and/or promoting attention (active wakefulness) in response to important stimuli, HA in sustaining general vigilance, and 5HT in maintaining quiet (non-attentive) wakefulness. While acetylcholine (ACh) does promote wakefulness, it appears to primarily function in mediating the sustained attention observed during “top-down” learning. The role of dopamine (DA) in sleep-wake state regulation is less clear, though DA neurons are known to engage in burst firing in response to rewarding and/or aversive stimuli, which promotes attention.

Sleep, wakefulness, and focused attention represent distinct brain patterns characterized by unique neuromodulator activity profiles and differing states of brain wave synchronization, frequency, and amplitude. During periods of focused attention (high alertness), brain electroencephalogram (EEG) recordings show desynchronized, high-frequency, low-amplitude patterns characterized by bursts of excitatory and inhibitory synaptic events across diverse brain areas. In contrast, EEG recordings during sleep display synchronized, low-frequency, high-amplitude oscillations (<1 Hz) as a result of alternation between firing and inactivity of large neuronal populations within brain regions (Lee and Dan, 2012). Neuromodulators play critical roles in modulating these brain states by effects largely on local interneuronal networks (Lei et al., 2007; Deng and Lei, 2008; Xiao et al., 2009; Salgado et al., 2012, 2016). NE from the locus coeruleus, 5HT from the raphe nuclei, HA from the tuberomammillary nucleus, and ACh from tegmental nuclei and basal forebrain are known to be involved in the regulation of sleep-wake states and have been implicated in the promotion of wakefulness, alterations in brain activity in response to stimuli (i.e., arousal, attention, etc.), and regulation of non-rapid-eye-movement (NREM) and rapid-eye-movement (REM) sleep (Eban-Rothschild et al., 2018; Figure 3).

NE, 5HT, and HA neurons are active during wakefulness, quiet during NREM, and silent during REM sleep (Boucetta et al., 2014; Yokoi et al., 2019). Locus coeruleus-NE neurons rapidly switch from tonic firing (1–3 Hz) during quiet wakefulness (Aston-Jones and Bloom, 1981b) to phasic burst firing (8–10 Hz) in response to salient stimuli (Aston-Jones and Bloom, 1981a) to enhance signal-to-noise and modulate frequency transmission (Aston-Jones and Bloom, 1981a,b; Bouret and Sara, 2005). NE neurons excite the reticular activating system while inhibiting neurons within sleep-active regions during wakefulness and/or in response to important stimuli (Brown et al., 2012). While HA neurons display similar arousal-state dependent activity to NE neurons (Takahashi et al., 2006), they primarily serve to maintain highly vigilant states (Fujita et al., 2017), whereas NE appears to play a larger role in the process of rapidly waking from sleep (Mitchell and Weinshenker, 2010) and responding to important/threatening stimuli. Tuberomamillary HA neurons are excited by 5HT (Eriksson et al., 2001) and NE (Brown et al., 2012), and in turn display excitatory effects on most elements of the reticular activating system (Brown et al., 2001; Haas et al., 2008). Similar to NE and HA neurons, 5HT neurons are also wake-active, and exhibit slow, tonic firing patterns across the wakeful states, with subpopulations demonstrating burst firing capabilities (Hajós et al., 2007). Unlike NE and HA neurons, most dorsal raphe-5HT neurons do not fire spontaneously and require afferent input from NE neurons to maintain their tonic output (Vandermaelen and Aghajanian, 1983). Also in contrast to NE and HA, 5HT neurons primarily promote quiet waking states (Jacobs and Fornal, 1991).

ACh and DA neuron activity is distinct from the monoaminergic members of the reticular activating system. While tegmental ACh neurons fire at high rates during wakefulness, *via* NE, 5HT, and HA neurons, they are also highly active during REM sleep (Lee and Dan, 2012), which is paradoxically characterized by a desynchronized EEG pattern, similar to the awake state. ACh projections from the basal forebrain are also involved in the regulation of brain activity states demonstrating similar firing patterns to tegmental nuclei but project primarily to the cortex (Brown et al., 2012) as opposed to the thalamus. Forebrain ACh neuronal output is believed to play a critical role in the mediation of sustained-attention, particularly within the context of knowledge-driven “top-down” learning (Sarter et al., 2001; Villano et al., 2017), and maybe attributable to improvements in the signal-to-noise ratio in cortical areas (Sarter et al., 2001; Picciotto et al., 2012). The role of DA in the regulation of brain activity states is less clear. DA neurons are well situated to contribute to wakefulness, as they display extensive reciprocal coupling with the wake-sleep regulatory network (Lu et al., 2006). Overall, DA neurons tend to maintain constant and stable firing patterns across various states (Steinfels et al., 1983), though ventral tegmental area-DA neurons engage in burst firing in the presence of salient rewarding and/or aversive stimuli (Brischoux et al., 2009; Cohen et al., 2012), highlighting their role in attention and arousal.

**Figure 4:**
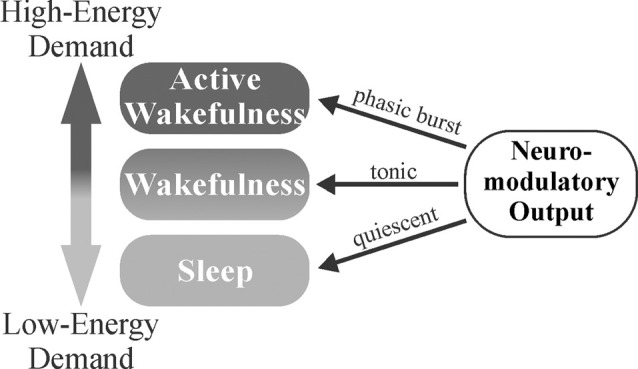
Neuromodulator-mediated gating of brain activity states may serve a homeostatic role. The balance of tonic vs. phasic firing may allow for the appropriate matching of environmental demands to the degree of attention. Less-metabolically demanding wakefulness is maintained during tonic firing with more metabolically demanding, desynchronized activity states gated behind phasic neuromodulatory bursts that trigger in response to salient stimuli. Decreased neuromodulatory tone during sleep (synchronized activity states) allows for clearance of metabolites that have accumulated during wakefulness.

The gating of brain activity state switching by neuromodulators may serve as a mechanism to conserve energy, maintain homeostasis, and affect circuit gain, particularly in sensory areas. When increased attention is required following exposure to important stimuli, neuromodulators engage in transient phasic burst firing that allows for rapid behavioral adaptation to changing environmental parameters (Aston-Jones and Bloom, 1981a; Takahashi et al., 2006; Ranade and Mainen, 2009). NE has been shown to suppress horizontal inputs in the visual cortex (Kobayashi et al., 2000), effectively enhancing the gain of extracortical visually-evoked inputs (Bouret and Sara, 2005). In the absence of such stimuli, slower, synchronized brain waves predominate. Sleep or synchronized states have been found to result in increased interstitial space fluid volume and metabolite clearance (Xie et al., 2013). Thus, the balance of tonic vs. phasic firing may allow neuromodulators to serve a homeostatic role, where less-metabolically demanding wakefulness is maintained during tonic firing, more metabolically demanding desynchronized activity states are gated behind phasic neuromodulatory bursts, and low neuromodulator output during sleep (synchronized activity states) allows clearance of accumulated metabolites (Figure 4). This system would allow an animal to respond quickly to important stimuli, preserve energy in the absence of threat/event, and clear accumulated toxins and metabolic waste products from the CNS during periods of slower, highly synchronized brain activity (*quiet wakefulness and sleep*). Overall, neuromodulators work synergistically to gate shifts in brain activity states for the accomplishment of differing tasks. NE, DA, and ACh are crucial for triggering desynchronized, high-activity states in response to various environmental stimuli (focus/attention and plasticity), while 5HT and HA primarily function in the general maintenance of wakefulness (Figure 3).

## Neuromodulators Shape Astrocyte Calcium Dynamics

Astrocytic calcium waves govern the communication between neuroglial networks. Recently, it has been suggested that neuromodulators affect astrocytic calcium transients and thereby modulate their output. 5HT is consistently demonstrated to increase fast, transient Ca^2+^ waves (Jalonen et al., 1997; Sanden et al., 2000; Schipke et al., 2011), while also inducing a secondary, longer-lasting oscillatory effect (Jalonen et al., 1997). Studies suggest that 5HT_2_ receptors appear to mediate these 5HT effects on astrocyte calcium (Jalonen et al., 1997; Sanden et al., 2000), potentially through PLC and IP_3_-mediated release of intracellular calcium stores (Jalonen et al., 1997). More specifically, in astrocyte-neuron co-cultures, 5HT was shown to increase astrocyte Ca^2+^ wave velocity while also decreasing the area of the wave (Blomstrand et al., 1999). NE, *via* α-1 adrenergic receptors, was also shown to stimulate calcium responses in astrocytes (Bekar et al., 2008; Ding et al., 2013; Paukert et al., 2014), most notably following direct locus coeruleus stimulation or startle *in vivo* (Bekar et al., 2008; Ding et al., 2013). It has also recently been demonstrated that NE primes astrocyte Ca^2+^ responses, lowering the threshold for subsequent responses to NE (Nuriya et al., 2017) as well as changing dynamics in response to synaptic glutamate (Muyderman et al., 2001; Paukert et al., 2014). ACh is suggested to act through both muscarinic and nicotinic receptors on astrocytes to induce calcium responses (Sharma and Vijayaraghavan, 2001; Takata et al., 2011). Evidence also exists for a DA-mediated increase in PLC-dependent Ca^2+^ mobilization in cultured astrocytes *via* D1 activation (Liu et al., 2009). Additionally, HA acts on H1 receptors to increase intracellular Ca^2+^ in astrocytes *in situ* (Shelton and McCarthy, 2000). Given the substantial evidence that neuromodulators impact astrocyte calcium dynamics, it follows that vast neuronal networks could be rapidly modulated in response to neuromodulator action on astrocytes.

Although most neuromodulators affect astrocyte calcium dynamics in some fashion, they do not all result in the same effect. There are two main anticipated outcomes of neuromodulator-mediated changes in astrocytic Ca^2+^: (1) altered K^+^ handling; and (2) gliotransmitter release. Neuromodulators affect astrocyte Ca^2+^ and this in turn influences K^+^ homeostasis (Wang et al., 2012b). As discussed above, alterations in K^+^ homeostasis are involved in regulating synaptic activity and are a potential mechanism through which neuromodulator-mediated action on astrocytes could affect synaptic function. The other major effect of neuromodulator induced astrocyte Ca^2+^ responses is the downstream effect this would have on gliotransmitter release. In support, 5HT_2B_ receptor activation promotes astrocytic ATP release (Kinoshita et al., 2018). NE is also associated with astrocyte ATP release that results in increased efficiency at glutamatergic post-synapses (Gordon et al., 2005). A recent HA study found that H1 activation increased Ca^2+^-dependent glutamate release, while H2 activation was associated with increasing cAMP (Kárpáti et al., 2018). DA, in a somewhat unique fashion to the other neuromodulators, is associated with increased glutamate gliotransmission when D2-A2A heterodimers are activated (Cervetto et al., 2017). Accordingly, neuromodulator-mediated effects on astrocyte Ca^2+^ provide compelling evidence for glial involvement in modulating synaptic function downstream of these signaling molecules.

## Neuromodulator-Mediated Effects on Cortical Inhibition

Consistent with their putative role in “brain-state” switching—regulating the sleep-wake cycle and gating periods of sustained attention—neuromodulators appear to filter corticocortical and thalamocortical information flow partly *via* modulation of cortical inhibition (Lei et al., 2007; Deng and Lei, 2008; Xiao et al., 2009; Salgado et al., 2012, 2016). Cortical inhibition refers to the process in which GABAergic interneurons attenuate the activity of cortical neurons in response to a variety of inputs (Daskalakis et al., 2007; Isaacson and Scanziani, 2011). This process is vital to the balanced interplay of excitation and inhibition observed in spontaneous cortical oscillations (Atallah and Scanziani, 2009) and the response to sensory stimuli (Monier et al., 2003; Wilent and Contreras, 2005; Isaacson and Scanziani, 2011). Appropriate interpretation of incoming sensory information is heavily reliant on the induction of inhibition (Aston-Jones and Bloom, 1981c; Bouret and Sara, 2005). Neuromodulator-induced alterations in cortical inhibition will therefore have a profound impact on the way sensory information is perceived. For example, neuromodulator-mediated tuning of local signal-to-noise ratio and frequency transmission, phenomena heavily influenced by cortical inhibition, is likely involved in the matching of local neuronal activity to the sensory demands of the environment; e.g., NE is released in a “burst-like” manner in response to salient stimuli (Aston-Jones and Bloom, 1981a), which amplifies the signal-to-noise ratio and improves the filtering of relevant vs. irrelevant information (Aston-Jones and Bloom, 1981a,b; Bouret and Sara, 2005).

The neuromodulators ACh, 5HT, and NE influence cortical inhibition across a variety of contexts to modulate the flow of sensory information. For example, during fear conditions, ACh activation of layer I interneurons leads to inhibition of layer II/III inhibitory interneurons, thereby diminishing inhibitory tone on pyramidal neurons (increased excitation; Letzkus et al., 2011). This process is central to associative fear learning in the auditory cortex, highlighting important functional roles for cortical inhibition. Regarding 5HT influence, we have recently demonstrated that 5HT increases spontaneous inhibition and attenuates evoked inhibition in the somatosensory cortex (Quon et al., 2018; Wotton et al., 2018), which suggests an important role for 5HT in modulating sensory adaptation. As for NE, it is proposed to influence cortical inhibition in a manner that improves signal-to-noise. Post-synaptically, NE depresses GABAergic influence through α1-receptors, which lowers the threshold for activation. In contrast, pre-synaptic α2- and β-receptor contributions increase GABA release probability (Salgado et al., 2012), which facilitates lateral inhibition through selective enhancement of perisomatic inhibition (Salgado et al., 2011). These effects of NE on cortical inhibition align with the known role of NE in “filtering” sensory information.

**Figure 5:**
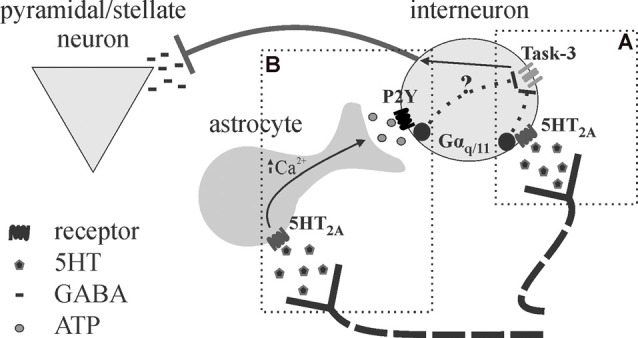
Proposed schematic for astrocytes as intermediary or amplifier of 5HT-mediated inhibition. **(A)** Deng and Lei (2008) show 5HT effects on inhibition in the auditory cortex are mediated by 5HT_2A_ receptors, Gα_q/11_, and task-3 potassium channel inhibition. **(B)** We recently provided evidence that astrocytes may be an intermediary in the effects of 5HT on cortical inhibition in the somatosensory cortex (Quon et al., 2018; Wotton et al., 2018), as the application of purinergic antagonists and disruption of astrocytic metabolism blocked the effects of 5HT. It appears possible that 5HT promotes astrocytic ATP release downstream of 5HT2A stimulation, leading to interneuron depolarization (P2Y also linked to Gα_q/11_). It is not clear whether astrocytes mediate 5HT effects entirely or merely serve to amplify the effects given their strategic position.

Neuromodulators, such as 5HT, may employ an astrocyte intermediary in the modulation of cortical inhibition (Figure 5). A study by Deng and Lei (2008) demonstrated a 5HT_2A_/G_q_-dependent depolarization of interneurons in response to 5HT, leading to increased spontaneous and decreased evoked inhibitory postsynaptic currents (sIPSCs; eIPSCs; Deng and Lei, 2008). However, as astrocytes express most 5HT receptors, and many P2Y receptors also link to G_q_ signaling pathways (Abbracchio et al., 2006; Erb et al., 2006), these findings do not rule out a role for 5HT-recruited astrocytic purinergic activity in cortical inhibition. We have recently demonstrated the effects of 5HT on cortical inhibition, characterized by increased spontaneous and reduced evoked inhibition, are blocked following the application of P2Y and A_2A_ antagonists (Quon et al., 2018; Wotton et al., 2018). This putative 5HT-induced purinergic signaling is likely astrocytic in origin, as disruption of astrocyte metabolism was found to impair 5HT responses on evoked inhibition (Quon et al., 2018; Wotton et al., 2018). The fact that disruption of astrocyte function and/or purinergic signaling blocks the effects of 5HT on spontaneous and evoked inhibition heavily implicates the involvement of astrocytes in 5HT-mediated cortical inhibition (Quon et al., 2018). Knowing that astrocytes are ideally situated to influence and/or synchronize activity across numerous neurons simultaneously, astrocytes may modulate multiple synapses *via* purinergic signaling to expand the range of 5HT effects on cortical inhibition.

## Neuromodulators Differentially Affect Extracellular Potassium Regulation

As mentioned above, alteration of [K^+^]_e_ is a potent means to affect neuronal and network activity. Astrocyte calcium-mediated uptake of K^+^ resulting in a mild, transient decrease in baseline [K^+^]_e_ is reported to increase the signal-to-noise ratio in the hippocampus (Wang et al., 2012b), whereas suppression of uptake resulting in a mild accumulation of baseline [K^+^]_e_ increases high-frequency oscillations (Ding et al., 2016; Bellot-Saez et al., 2018; Rasmussen et al., 2019) as well as gain in the visual cortex (Rasmussen et al., 2019). Recent modeling using the Averaged-Neuron computational model demonstrated [K^+^]_e_ to be a potent mediator of changes in brain state, although calcium and magnesium also play a role (Rasmussen et al., 2017). Using this computational model they showed that, although changes from sleep to wakefulness required inhibition of calcium-sensitive potassium channels with changes in extracellular ions only affecting the threshold, the transition from quiet to active wakefulness was mediated by a subtle change in extracellular ions; K^+^ being the most potent (Rasmussen et al., 2017). Furthermore, given the known ability for neuromodulators to inhibit the calcium-sensitive K^+^ channel (McCormick and Williamson, 1989; McCormick et al., 1993) and affect baseline [K^+^]_e_ (Ding et al., 2016; Wotton et al., 2020), it is reasonable that an increase in tonic neuromodulator release and effects on calcium-sensitive K^+^ channels induces general wakefulness whereas phasic neuromodulator release with associated effects on extracellular ion concentrations governs transitions between quiet and active wakefulness.

Modulation of astrocyte-mediated K^+^ uptake and distribution can occur *via* three distinct pathways. The first involves a synaptic activity-mediated increase in astrocyte [Na^+^] *via* glutamate transport to drive the Na^+^/K^+^ ATPase (Larsen et al., 2016b). The second involves an astrocyte calcium-mediated increase in [Na^+^] *via* the Na^+^/Ca^2+^ exchanger (Wang et al., 2012b) that may or may not depend on neural activity. Finally, the third involves direct long-range neuromodulator-mediated regulation of Na^+^/K^+^ ATPase or Kir4.1 independent of local network activity (Wotton et al., 2020). Such diversity over the control of [K^+^]_e_ regulation that may or may not depend on local synaptic activity enables a powerful means to regulate brain-wide network connectivity and, ultimately, behavior.

**Figure 6:**
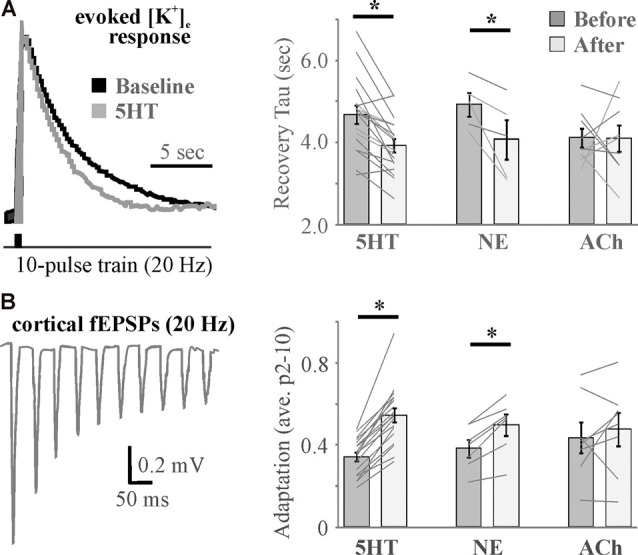
Differential effects of neuromodulators on evoked increases in extracellular potassium mirror differential neuromodulator effects on adaptation. **(A)** Example traces (left) and histograms (right) showing activity-induced recovery decay tau is accelerated by 5HT and NE, but not ACh. **(B)** A 10-pulse train shows significant spike frequency adaptation in the somatosensory cortex (left) that is differentially regulated by neuromodulators (right). *N* = 5–18. * <0.05 by repeated-measures ANOVA with Fisher’s LSD *post hoc*. Data and figures were adapted from Wotton et al. (2020) with permission.

Although a cocktail of neuromodulators (NE, ACh, DA, orexin, and HA) induced an increase in [K^+^]_e_ that was associated with the transition in brain state (Ding et al., 2016), recent evidence suggests that individual neuromodulators have differential effects (Wotton et al., 2020). 5HT, NE, and ACh differentially affected inward rectifiers and the Na^+^/K^+^ ATPase to exert distinct effects on baseline [K^+^]_e_ as well as recovery from evoked K^+^ increases that were associated with effects on somatosensory adaptation (Figure 6) and amplitude (Wotton et al., 2020). 5HT was found to affect K^+^ regulation and somatosensory adaptation *via* effects on Kir, whereas NE and ACh effects were mediated by opposing action on Na^+^/K^+^ ATPase activity. Thus, in addition to direct differential action on neurons and probable differences in effects on astrocyte gliotransmission, neuromodulators differentially affect astrocyte regulation of [K^+^]_e_ to expand the repertoire by which neuromodulators govern network activity and connectivity.

## Conclusion

Given their sponge-like morphology and positioning, the hypothesis that astrocytes extend neuromodulator effects would appear to rectify the gap between the limits imposed by simple diffusion and the rapid, far-reaching influence of neuromodulators on brain-state transitions. Despite being released extrasynaptically in a volume manner, neuromodulator effects are limited by diffusion in the complex CNS. This manner of unaided diffusion appears to be an inefficient method to alter networks/synapses in the widespread and timely manner known to be characteristic of neuromodulator signaling. Intriguingly, astrocytes express receptors for neuromodulators, form a far-ranging interconnected syncytium, demonstrate calcium-wave propagation in response to neuromodulator binding, influence the function of a multitude of synapses through the release of gliotransmitters and regulation of extracellular potassium, and can promote synchronicity of neuronal populations that are related to the different brain states. Perhaps the different brain states can be viewed as a means to conserve energy with neuromodulators (and astrocytes) gating desynchronized up-states and circuit gain only in times of need, while synchronized slow oscillations (down-states) reduce energy demands and help clear the brain of wastes and metabolites. Astrocytes are necessary both for eliciting neuromodulator effects and extending their influence/reach beyond that of simple diffusion. We conclude that astrocytes are ideally positioned and suited to be the ultimate effectors of long-range neuromodulatory networks.

## Author Contributions

All authors listed have made a substantial, direct and intellectual contribution to the work, and approved it for publication.

## Conflict of Interest

The authors declare that the research was conducted in the absence of any commercial or financial relationships that could be construed as a potential conflict of interest.
